# Effects of multiple environmental variables on tundra ecosystem respiration in maritime Antarctica

**DOI:** 10.1038/s41598-018-30263-6

**Published:** 2018-08-17

**Authors:** Tao Bao, Renbin Zhu, Xianglan Li, Wenjuan Ye, Xiao Cheng

**Affiliations:** 1 Institute of Polar Environment & Anhui Key Laboratory of Polar Environment and Global Change, School of Earth and Space Sciences, University of Science and Technology of China, Hefei, 230026 China; 2 College of Global Change and Earth System Science, Beijing Normal University, Beijing, 100875 China

## Abstract

Summertime ecosystem respiration (ER) rates through seven sites were measured at an upland tundra on Fildes Peninsula in maritime Antarctica to investigate effects of topographic gradient, vegetation types and climatic factors on tundra ER rates. Overall the highest ER rates occurred at the tundra top, followed at the middle slope, and the lowest rates at the lower slope. The daily highest ER rates occurred at noon whereas the lowest at 6 am. There was a significant positive correlation (P < 0.05) between tundra ER and 0–10 cm soil temperature, but a significant negative correlation (P < 0.01) between ER and soil moisture. A high Q_10_ value of 2.69 was obtained when all the data were combined, indicating soil temperature sensitivity of tundra ER. The mean ER at the tundra sites with moss coverage (72.2 ± 4.4 mg CO_2_ m^−2^ h^−1^) was significantly higher (P < 0.01) than that at the sites with lichen coverage (46.8 ± 8.7 mg CO_2_ m^−2^ h^−1^). The tundra sites without snow coverage experienced significant CO_2_ release, whereas the emission through ER was very low at the tundra sites with snow coverage. Our results indicated that topographic gradient, soil temperature, soil moisture, vegetation types and snow coverage might affect tundra ER in maritime Antarctica.

## Introduction

Carbon dioxide (CO_2_) is key active greenhouse gas (GHG) contributing to global warming^1^. Increasing atmospheric concentration of CO_2_ has stimulated research on its emission from both terrestrial and aquatic environments^1^. Ecosystem respiration (ER), as a key component of global carbon cycle, is one of important sources for atmospheric CO_2_. At present, the CO_2_ emissions through ecosystem respiration have been extensively investigated from temperate, subtropical and tropical terrestrial ecosystems, and they are affected by the geographical differences at the global scale^1–3^. Polar Regions would be the most vulnerable to climate change in the world, and numerous researches about effects of climate warming on CO_2_ fluxes have occurred in Arctic tundra ecosystems^4–7^. The studies about CO_2_ emissions from Antarctic terrestrial ecosystem mainly concentrated on the McMurdo Dry Valleys of continental Antarctica^8–10^. However, only few data about CO_2_ emissions are available from maritime Antarctic tundra according to the limited references^11–13^.

To better understand the relationships of CO_2_ emissions to environmental parameters, ER rates and net CO_2_ fluxes have been measured at a number of sites of boreal or Arctic region and some sites of Antarctic region for the past three decades. Soil physicochemical properties, soil microbial community structure, and vegetation coverage are important factors affecting the ecosystem carbon balance and ER in Arctic and sub-Arctic areas^4,5^. Higher CO_2_ emissions from the tundra are predicted to become a positive feedback to climate warming as a result of increased soil organic matter decomposition and consequent higher nutrient availability^6^. Soil moisture directly affects vegetation distribution and functioning, and controls microbial decomposition influencing soil respiration, and thus it has both direct and indirect effects on CO_2_ exchange in tundra ecosystems^14^. Vegetation composition and productivity have a direct effect on tundra ER^10^. Significantly weak and spatially variable CO_2_ emissions were found in the McMurdo Dry Valleys, which are located in continental Antarctica, due to low soil organic carbon contents, and they were driven mainly by soil temperature and moisture, and their interaction with biological elements^8–10,15^. However, the results from Arctic regions or the McMurdo Dry Valleys of continental Antarctica might not be applied to maritime Antarctic tundra due to differences in vegetation communities, soil nutrients and climatic conditions. The available works are few, and more data about tundra CO_2_ emissions are needed in maritime Antarctica^11–13^. Therefore it is very necessary to conduct the research about CO_2_ emission from the tundra in maritime Antarctica.

In maritime Antarctica, the response of tundra ecosystem is sensitive to climate warming, accompanied by the regional changes in precipitation^16,17^. Warming climate might cause several consequences in the tundra ecosystem, especially in soil formation and the spatial extent of expanding vegetation^12^. The most common moss vegetation (*Bryum Pseudotriquetrum and Bryum muelenbeckii*) and native vascular plant species (*Colobanthus quitensis and Deschampsia antarctica Desv*) have recently been expanding in maritime Antarctic tundra^16,18–20^. These changes would affect tundra ER rates, and net CO_2_ fluxes since tundra vegetation composition and soil physical properties are related to soil organic carbon content, potential mineralization and microorganism activity^4,21,22^. Therefore, the measurements of ER rates are essential to understand the C cycling dynamic in the maritime Antarctic tundra, and their feedbacks to the regional warming climate^12^. Summertime CH_4_, N_2_O and CO_2_ fluxes, and ER rates have also been *in situ* observed from various tundra ecosystems in maritime Antarctica^13,19,23–25^. The increasing temperatures might decrease tundra CO_2_ sink accompanied by the increase in soil respiration^13,19^. Tundra soil and vegetation are closely linked to the global carbon cycling dynamics, especially CO_2_ exchange in maritime Antarctica^11–13^. More data about tundra ER are required to improve our knowledge about CO_2_ exchange.

During the austral summer of 2014/2015, the ER rates at seven sites were measured in an upland tundra on Fildes Peninsula, maritime Antarctica. The objectives of this study were (1) to study summertime and daily variability in tundra ER; (2) to investigate effects of topographic gradient on tundra ER; (3) to examine effects of soil temperature, soil moisture, snow coverage and vegetation types on tundra ER. This is an attempt to increase the tundra ER data set in order to reasonably estimate carbon budget, and to find out environmental variables affecting ER in maritime Antarctica.

## Results

### Climate and tundra soil characteristics

**Figure 1:**
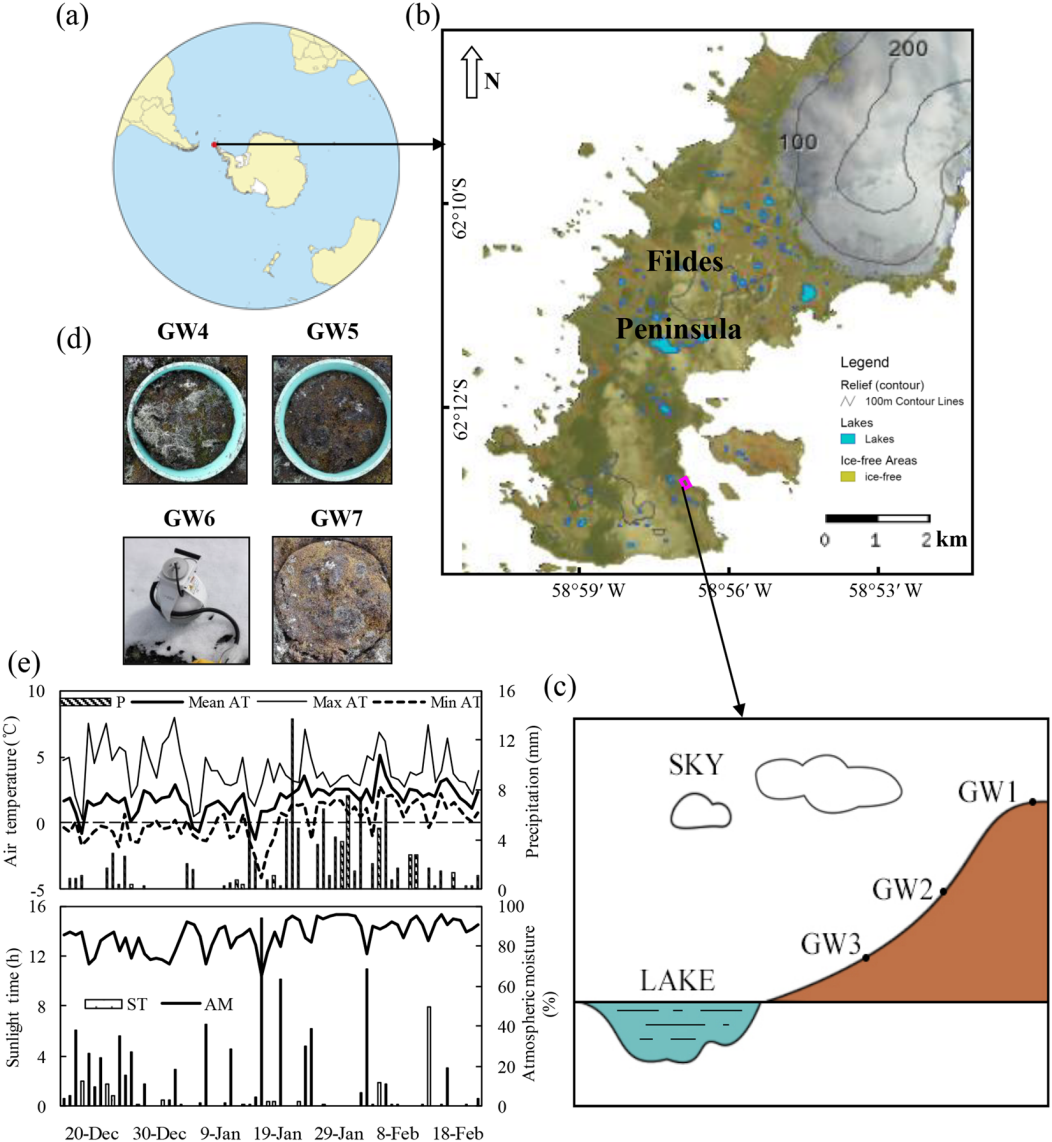
(**a**) The dot indicates location of the investigation area in maritime Antarctica. (**b**) Location of the study sites in Fildes Peninsula. (**c**) The position of the investigation sites GW1, GW2 and GW3 in upland tundra of Fildes Peninsula; (**d**) The background of the sites GW4, GW5, GW6 and GW7; (**e**) The climate conditions during tundra ER measurements. Note: The map was drawn using CorelDRAW 2017 (http://www.corel.com/cn/) and Microsoft Excel 2016 (https://products.office.com/zh-cn/excel) software.

**Figure 2:**
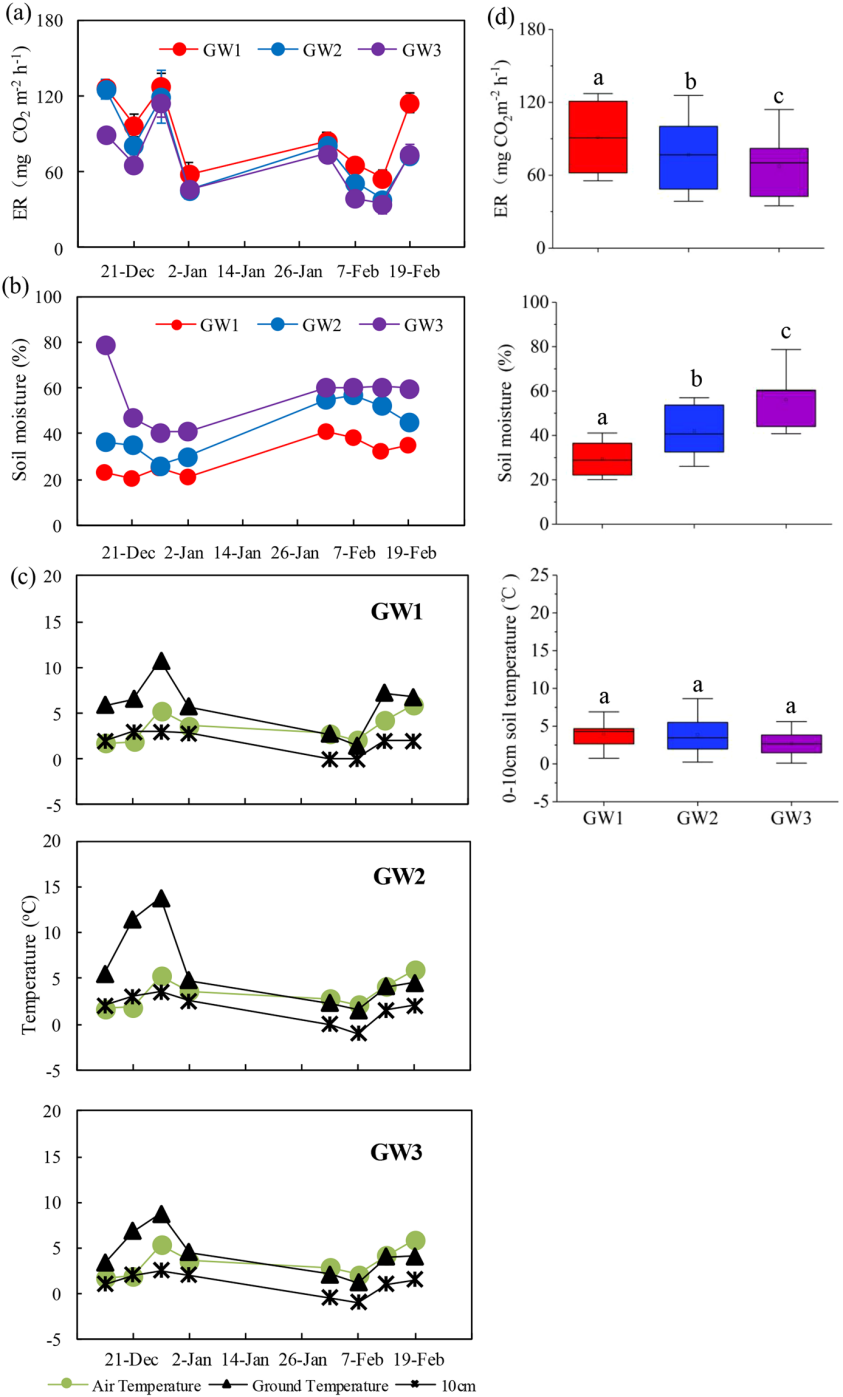
The summertime ER (mg CO_2_ m^−2^ h^−1^ ± SE, n = 3) along the topographic gradient at an upland tundra. Panel (a) ER, (**b**) soil moisture, (**c**) air and soil temperature, and (**d**) The comparisons of the ER, soil moisture and soil temperature between the sites GW1, GW2 and GW3. Note: GW1, GW2 and GW3 were located at the tundra top, the middle slope and at the lower tundra slope. Error bar indicates standard error of the means (n = 3). Boxes enclose the interquartile range, whiskers show the full range. The different lowercase letters indicate statistically significant differences between the means (Fisher’s LSD, P < 0.05).

During the austral summer of 2014/2015, the mean air temperature (AT) showed a small increase from Dec 20, 2014 to Feb 18, 2015, and the daily minimum AT was often below 0 °C while the maximum AT was generally above 3 °C (Fig. 1). The total precipitation was 114 mm, and total sunlight time (ST) was 117 h. The air temperature, ground temperature (GT) and 10 cm soil temperature (ST_10_) showed a similar change at the sites GW1, GW2 and GW3, and they increased gradually from December, 2014 to January, 2015, but soil temperatures decreased with soil depths (Fig. 2). The mean AT, GT and ST_10_ were 3.4 ± 0.6 °C, 5.9 ± 1.0 °C and 1.5 ± 0.3 °C, respectively.

Physiochemical properties of the tundra soils were summarized in Table 1. The mean soil moisture (SM) decreased from the lower slope (59.0%) to the top of tundra (30.4%). The TOC contents and C/N ratios were almost equal to each other in all the soil samples, ranging from 6.7 to 7.1% and from 9.6 to 10.3, respectively, and the tundra soils were neutral with the pH of 6.8–7.3. However, soil TN, NH_4_^+^-N, NO_3_^−^-N and TS contents were highly variable from the lower slope to the top of tundra due to environmental heterogeneity. The mean soil TN and TS contents ranged from 0.23 to 0.32 mg g^−1^ and from 0.07 to 0.15 mg g^−1^, respectively. The mean soil NH_4_^+^-N and NO_3_^−^-N concentrations increased from the lower slope to the top of the tundra.

**Table 1:** Soil properties and plant species at the sites for the upland tundra in maritime Antarctica.

Tundra Sites	Soil Properties							Characteristic plant species
	SM (%)	pH	TOC (%)	NH_4_^+^-N (μg g^−1^)	NO_3_^−^-N (μg g^−1^)	C/N	S (mg g^−1^)	
GW1	30.4	7.3	6.86	0.62	0.33	9.7	0.15	*Bryum pseudotriquetrum, Bryum muelenbeckii, Sanionia uncinata, Warnstorfia sarmentosa, Usnea sp*.
GW2	45.7	7.1	6.96	0.14	0.17	9.6	0.13	
GW3	59	6.8	6.66	0.06	0.19	10	0.08	
GW4	42.8	6.9	6.67	0.24	0.19	10.3	0.07	
GW5	44.9	7	6.98	0.16	0.16	9.6	0.12	
GW6	54.6	7.2	6.96	0.22	0.18	9.7	0.12	
GW7	47	7.2	7.12	0.24	0.22	9.6	0.14	

Note: SM, TOC and C/N indicated soil moisture, total organic carbon and the ratios of soil organic carbon and nitrogen, respectively.

### Summertime tundra ER along the topographic gradient

During the observation period, tundra ER rates showed a similar fluctuation at the tundra top site GW1, the middle slope site GW2, and the lower slope site GW3. All the three sites experienced a significant release of CO_2_ (Fig. 2). The ER rates ranged from 55.4 to 127.2 mg CO_2_ m^−2^ h^−1^ at GW1, from 38.5 to 125.7 mg CO_2_ m^−2^ h^−1^ at GW2, and from 34.8 to 114.0 mg CO_2_ m^−2^ h^−1^ at GW3, respectively. The ER rates showed a significant difference (P < 0.05) between the sites GW1, GW2 and GW3. Overall the highest mean ER rate occurred at GW1 (mean 91.2 ± 10.6 mg CO_2_ m^−2^ h^−1^), followed at GW2 (mean 76.9 ± 11.4 mg CO_2_ m^−2^ h^−1^), and the lowest at GW3 (mean 67.3 ± 9.5 mg CO_2_ m^−2^ h^−1^) (Fig. 2d).

**Figure 3:**
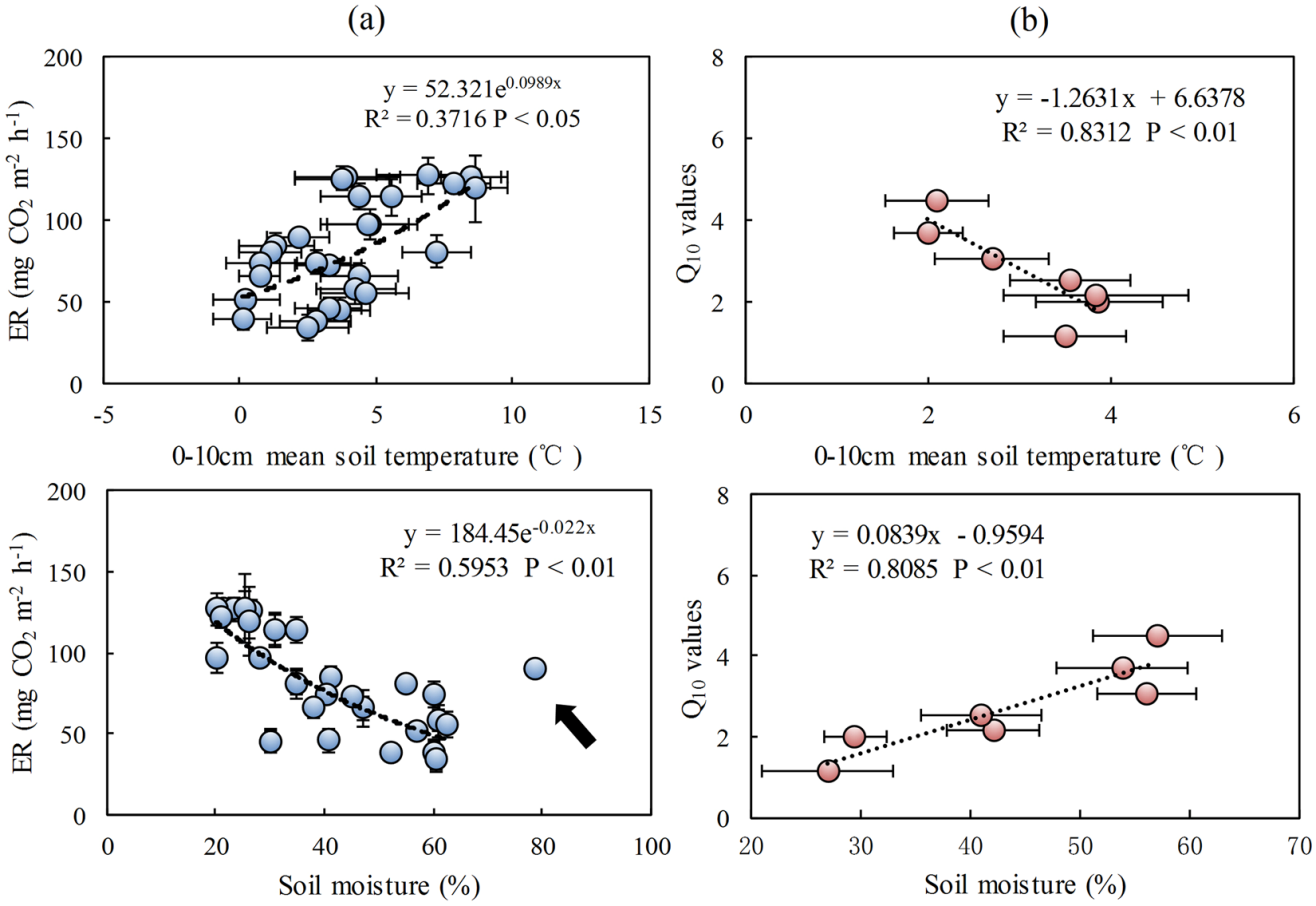
Panel (a) Relationship between ER and 0–10 cm mean soil temperature, soil moisture at the tundra sites. (**b**) Correlations between tundra Q_10_ values and soil temperatures, soil moisture. The arrow indicates outlier data, which are excluded from the correlation. Note: the figures for Q_10_ fitting at each site were given in Supplementary Material.

Tundra ER showed a similar variation with air temperature, ground temperature and 10 cm soil temperature during the observation period (Fig. 2c). Overall, tundra ER rates were significantly affected by soil temperature (F = 3.67, P < 0.05), soil moisture (F = 6.36, P < 0.01) and their interaction (F = 3.49, P < 0.05) (Table 2). In addition, there was a significant exponential correlation (R^2^ = 0.37, P < 0.05) between tundra ER and 0–10 cm mean soil temperature. Tundra ER showed a significant negative correlation (R^2^ = 0.60, P < 0.01) with soil moisture (Fig. 3a). Tundra ER can be more accurately predicted using a bivariate linear model that incorporates both soil temperature and moisture as independent variables (Table 3). A high Q_10_ value of 2.69 was obtained, indicating soil temperature sensitivity of summertime ER in maritime Antarctic tundra. The Q_10_ showed a negative correlation (R^2^ = 0.83, P < 0.01) with tundra soil temperatures, but a significant positive correlation (R^2^ = 0.81, P < 0.01) with soil moisture based upon a linear regression model (Fig. 3b). No significant correlation (P > 0.05) was found between tundra ER and soil chemical properties (TOC, NH_4_^+^-N, NO_3_^−^-N, C: N) at all the observation sites although the differences in the emission rates might reflect the ecological and environmental heterogeneity at the different tundra sites.

**Table 2:** Tests of significance of date (DA), soil temperature (ST), soil moisture (SM), vegetation types (VT), snow coverage (SC) and their interactions on the tundra ER using multivariate ANOVA (F and P values).

	F	P
**GW1–3 Combined (n = 24)**		
DA	12.06	<0.001^c^
ST	3.67	<0.05^a^
SM	6.36	<0.01^b^
ST*SM	3.49	<0.05^a^
**GW4–5 Combined (n = 18)**		
DA	17.84	<0.001^c^
ST	2.99	0.112
SM	10.37	<0.01^b^
VT	22.70	<0.001^c^
ST*SM	0.25	0.627
ST*VT	2.23	0.147
SM*VT	6.06	<0.01^b^
ST*SM*VT	2.62	0.114
**GW6–7 Combined (n = 18)**		
DA	4.26	<0.05^a^
ST	21.77	<0.001^c^
SM	5.49	<0.05^a^
SC	28.10	<0.001^c^
ST*SM	5.21	<0.05^a^
ST*SC	24.50	<0.001^c^
SM*SC	15.16	<0.001^c^
ST*SM*SC	7.09	<0.01^b^
**GW1–7 Combined (n = 60)**		
ST	9.03	<0.01^b^
SM	6.63	<0.01^b^
VT	7.35	<0.01^b^
SC	22.96	<0.001^c^
ST*SM	5.56	<0.01^b^
ST*SM*VT	1.70	0.193
ST*SM*SC	4.67	<0.05^a^
ST*SM*VT*SC	1.03	0.316

Note: Superscripts a, b and c indicate significant effects at P < 0.05, 0.01 and 0.001, respectively.

**Table 3:** Correlations of summertime soil respiration rates and soil moisture, 0–10 cm mean soil temperature at the tundra sites of the upland tundra.

Sites	Equation		
	F_ER_ = aW + bT + c	R^2^	n
GW1	F_ER_ = 47.44-0.42 W + 8.11 T	0.46	8
GW2	F_ER_ = 78.88-0.45 W + 4.46 T	0.49	8
GW3	F_ER_ = 25.46-1.05 W + 12.59 T	0.42	8
GW1–3 Combined	F_ER_ = 67.60-0.24 W + 6.11 T	0.54	24

Note: Only the equations and R^2^ values significant at p < 0.05 are shown. F_ER_: Soil CO_2_ respiration rates; W: Soil moisture; T: 0–10 cm mean soil temperature.

### Diel tundra ER

**Figure 4:**
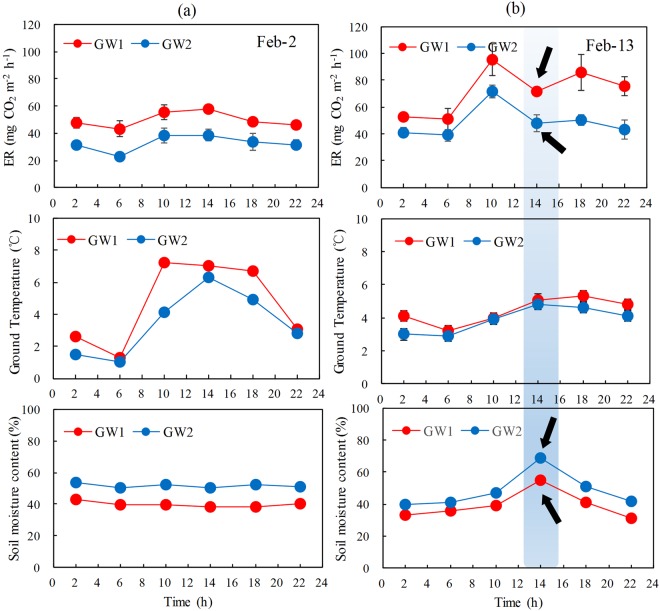
Diurnal variations of tundra ER, soil temperature and soil moisture at the tundra sites GW1 and GW2.

The ER showed a similar diurnal pattern at the top site GW1 and the slope site GW2 on Feb 2, 2015. The highest emission rates both occurred at noon (10 am to 14 pm), and the lowest at 6 am (Fig. 4a). The daily mean ER rate at GW1 (49.9 mg CO_2_ m^−2^ h^−1^) was significantly higher than that at GW2 (32.8 mg CO_2_ m^−2^ h^−1^). On Feb 13, the ER rates also showed a similar diurnal pattern at GW1 and GW2, with the lowest ER at 6 am. However, the abnormal low ER rates occurred at 2:00 pm, corresponding to significantly enhanced soil moisture (Fig. 4b). The sudden precipitation took place at that moment, thus high soil water content could suppress soil CO_2_ release. The CO_2_ emission rates ranged from 51.1 to 95.0 mg CO_2_ m^−2^ h^−1^ at GW1 and from 40.0 to 69.0 mg CO_2_ m^−2^ h^−1^ at GW2, and mean emission rate (72.0 ± 7.2 mg CO_2_ m^−2^ h^−1^) at GW1 was also significantly higher than that at GW2 (49.0 ± 4.9 mg CO_2_ m^−2^ h^−1^). Calculated from 6 individual measurements over 24 h in upland tundra, ER rates between 6:00 and 10:00 were close to the mean diurnal tundra ER.

The daily ER rates showed a similar trend to ground temperatures, and two ER peaks coincided with those of ground temperatures, indicating that high temperature increased tundra ER. Tundra ER showed the lowest values in the morning corresponding to the lowest ground temperature (Fig. 4). The soil temperatures show a large fluctuation at GW1 and GW2, whereas the fluctuation of soil moisture was small, and almost stable. Overall soil temperature was higher, but soil moisture was lower at GW1 than at GW2.

### Tundra ER under different vegetation coverage

**Figure 5:**
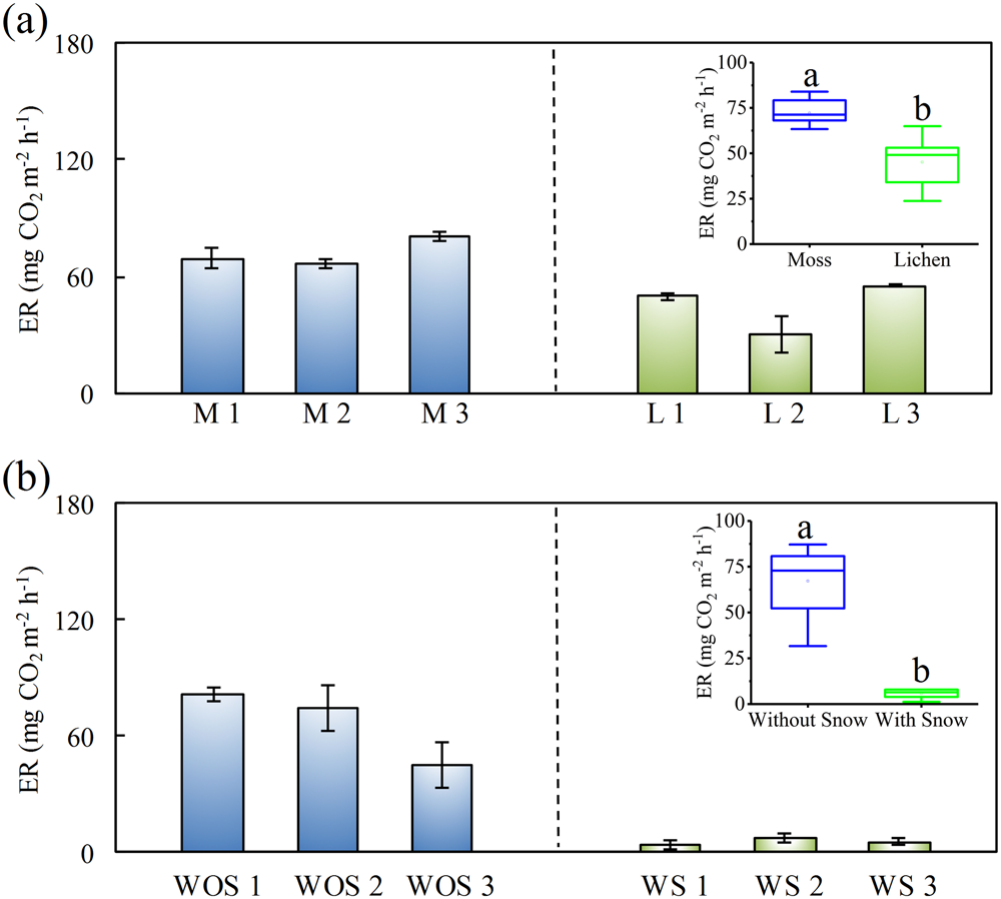
Effects of (**a**) vegetation types and (**b**) snow coverage on ER rates at the upland tundra sites. M 1–3 and L 1–3 indicated the sub-sites with moss coverage and with lichen coverage, respectively. WOS 1–3 and WS 1–3 indicated the sub-sites without snow coverage and with snow coverage, respectively. Boxes enclose the interquartile range, whiskers show the full range. The different lowercase letters indicate statistically significant differences between the means (Fisher’s LSD, P < 0.05).

The tundra ER rates at lichen-covered (GW4) and moss-covered (GW5) tundra sites ranged from 23.8 to 64.9 mg CO_2_ m^−2^ h^−1^, and from 63.4 to 84.0 mg CO_2_ m^−2^ h^−1^, respectively. The mean ER at GW5 (three subsites, n = 9, 72.2 ± 4.4 mg CO_2_ m^−2^ h^−1^) was significantly higher (P < 0.01) than that at GW4 (three subsites, n = 9, 46.8 ± 8.7 mg CO_2_ m^−2^ h^−1^), indicating that moss-covered tundra soil could release more CO_2_ than lichen-covered tundra soil (Fig. 5a). For the two sites, ER rates were significantly affected by soil moisture (F = 10.37, P < 0.01), vegetation types (F = 22.70, P < 0.001) and their interaction (F = 6.06, P < 0.01) (Table 2), but no significant change was affected by soil temperature (Table 2), although ER rates increased with soil temperature (Fig. S1).

### Tundra ER under snow coverage

The ER rates showed a large difference between tundra sites with snow coverage (GW6) and without snow coverage (GW7) (Fig. 5b). The site GW7 experienced a significant CO_2_ release with the mean emission rate of 67.2 ± 11.1 mg CO_2_ m^−2^ h^−1^ (three subsites, n = 9), whereas the ER rates at the site GW6 was almost extremely low (three subsites, n = 9). The ER rates at the tundra sites without snow coverage was significantly higher (P < 0.01) than those at the sites with snow coverage, indicating that accumulated snow could greatly limit ecosystem respiration or prevent CO_2_ emissions from the tundra ecosystem. Overall, snow coverage had a significant effect (F = 28.10, P < 0.001) on tundra ER rates, and the interaction among soil temperature, soil moisture and snow coverage on ER rates (F = 7.09, P < 0.01) was also observed during the observation period (Table 2).

## Discussion

Previous climate model simulations have shown that both annul and seasonal responses of terrestrial carbon cycle to climate change will change in the 21th century, but with large uncertainty at high latitudes (Antarctic and Arctic)^26,27^. This study is therefore valuable for the research of terrestrial carbon cycle modelling and the comparisons of mean CO_2_ emission rates within different Antarctic regions could approximately reflect the differences of ER. In our study area, the mean CO_2_ emission (46.8–91.2 mg CO_2_ m^−2^ h^−1^) at upland tundra sites was larger than those measured for soils in the Taylor Valley (15.8–23.8 mg CO_2_ m^−2^ h^−1^)^15^, at Rothera Point of Marguerite Bay (23.8–30.1 mg CO_2_ m^−2^ h^−1^), and at Anchorage Island (25.3–33.3 mgCO_2_ m^−2^ h^−1^)^12^, and close to those (26.4–124.1 mg CO_2_ m^−2^ h^−1^) measured on lakeshore soils in Garwood Valley, Antarctica^8^. In addition, our results were comparable to those measured at a seal colony (70.8 ± 7.6 mg CO_2_ m^−2^ h^−1^) and the tundra sites adjacent to a penguin colony^13^. However, the mean ER rates in the study area were almost one tenth of those measured at penguin colony tundra (201.3 ± 31.4 mg CO_2_ m^−2^ h^−1^)^13,19^. Higher soil organic carbon level at penguin colony than our study area might provide the favorable environment for microbial activity, resulting in enhanced soil respiration^13,19^. Hopkins *et al*.^28^ found that the composition and mineralization of lacustrine detritus and soil organic matter affected soil respiration in Antarctic Garwood Valley soils. However, soil chemical properties were similar to each other at our sites, and no significant correlation (P > 0.05) was found between tundra ER and soil TOC, other chemical properties at all the observation sites in our study area.

Overall the highest ER rates occurred at the tundra top, followed at the middle slope, and the lowest emission rate at the lower slope (Fig. 2), indicating that topographic gradient might have an important effect on spatial variability in tundra ER. Topography is the primary determinant of soil moisture patterns across arctic landscapes and plays a major role in determining the distribution of vegetation types and plant community succession^29^. The variation in plant biomass, rates of primary productivity, soil organic matter (SOM) quantity and quality, nutrient status and soil chemistry are all tightly linked to topography^30^. In total, topography results in spatial variations in environmental variables through establishing elevation gradients, and in turn influences the rates of tundra carbon transformation processes. In our study area, soil temperature increased with the elevation from GW3 to GW1, but soil moisture significantly decreased with the elevation. Our results indicated that topography gradient plays critical roles through their influences on soil moisture, soil temperature and their interaction that in turn affect ER and C biogeochemical cycling in the tundra ecosystems of maritime Antarctica.

On the other hand, corresponding topographical gradients of soil moisture and temperature tend to occur at the landscape and larger spatial scales across the Arctic^31^. In maritime Antarctica, tundra ecosystems generally developed on the elevated, hilly and uneven upland along the coast. Faster rates of organic matter mineralization and decomposition (i.e. CO_2_ emissions) occur in inter-hummock areas^13,30^. In this study, higher soil moisture and lower temperature occurred at the slope site GW3 which might limit soil organic matter decomposition by microorganisms, resulting in soil organic matter accumulation in low-lying areas^30^. Therefore topography is a key factor for the evaluation of tundra ER at the tundra landscape and larger spatial scales in maritime Antarctica.

The temporal variation range of ER (34.9 to 127.2 mg CO_2_ m^−2^ h^−1^) was considerable as tundra ER varied with soil temperatures (Fig. 2). The ER rates showed a similar diurnal pattern at the top site GW1 and the slope site GW2, corresponding to ground temperature (Fig. 4). It was demonstrated that there was a diurnal cycle both of tundra photosynthesis and respiration in the Antarctic Peninsula^12^, and our results corroborated previous research. Overall the ER rates showed a significant positive correlation (P < 0.05) with soil temperatures in maritime Antarctic tundra (Fig. 3a). The activities of soil or plant enzymes and soil microorganisms were gradually enhanced with the increase in soil temperatures, and then led to increased ecosystem respiration rates, whereas the low temperature would restrict the respiration of the vegetation and soil microorganisms, thus reduced tundra ER rates^9,12^. Similarly, the ER generally showed a strong positive correlation with soil temperature in Arctic tundra, and climate warming might decrease CO_2_ sink through increase in tundra ER^9,32^. In McMurdo Dry Valley of east Antarctica, several studies also showed a similar correlation between ER and soil temperatures^10,33^. Overall ER indicated greater temperature sensitivity in Polar Regions than other global regions. The faster increase in temperatures have occurred in maritime Antarctica compared with other global regions in the past few decades^17^. The positive correlation of ER with soil temperature might reflect the positive feedback of tundra ecosystem respiration to current warming climate in maritime Antarctica.

A high Q_10_ value (Q_10_ = 2.69) showed soil temperature sensitivity of summertime ER in maritime Antarctic tundra. The Q_10_ values for soil respiration ranged from 2.00 to 3.00 on Antarctic Peninsula, and from 2.00 to 4.40 in the Garwood Valley, east Antarctica^28^. Raich & Schlesinger^2^ reported a Q_10_ range of 1.30–3.30 for different biomes of the world. Our observed Q_10_ values were within the high results reported in the references. Prior research suggested that Q_10_ could vary according to the range of temperatures used for the calculation, and highlighted that it could be subjected to inter-annual and seasonal variations^34^. In this study, the Q_10_ values showed a negative correlation with tundra soil temperatures (Fig. 3b). Generally the Q_10_ values for ER decreases with the increase in soil temperature^2,34^. The Q_10_ dependence on temperature might be related with the response of soil microorganisms or soil enzymatic activities to different temperature ranges^2,34,35^. Significant exponential correlations between tundra ER and soil temperatures (Fig. 3a) indicated that climate warming might decrease tundra CO_2_ sink through increase in soil respiration, thus affect tundra CO_2_ exchange in maritime Antarctica.

A significant negative correlation (P < 0.01) between ER and soil moisture (Fig. 3a) indicated that soil moisture might have an important effect on ER rate. Similarly, a weak negative correlation between soil respiration and soil moisture was found in the McMurdo Dry Valleys, east Antarctica^10,33^. Soil moisture directly affected tundra vegetation distribution and functioning, and played major roles in structuring soil microbial communities by constraining their physiology and distribution and the chemical substrates used by soil microorganisms^28^. High soil water content might prevent atmospheric oxygen from diffusing into the soil, and decreased the ecosystem respiration^4^. On the contrary, the drying of near-surface soil layers and consequent increased diffusion of soil gases might allow more rapid efflux of CO_2_ emissions to the atmosphere^36^. In this study, the diel variation of ER was also constrained by soil moisture, and the abnormal low ER occurred at 2:00 pm (Fig. 4b), corresponding to significantly enhanced soil moisture due to the heavy precipitation. Similarly, the diel variation in ER was largely controlled by soil moisture in a desert shrub ecosystem^35^. Therefore the increase in soil moisture might have a significant impact on C and N mineralization^10,33^, and further inhibited CO_2_ exchanges between tundra ecosystem and atmosphere in maritime Antarctica.

The multivariate regression analysis indicated that about 70% of the variation in ecosystem respiration rate was jointly affected by soil temperature and soil moisture^4^. Generally it was difficult to independently differentiate the effects of soil temperature and soil moisture under field condition due to the hydrothermal interaction^34,35^. When the soil temperature increased to 10–20 °C, effects of soil moisture on ER rate gradually increased, whereas soil moisture had little effect on ecosystem respiration when the soil temperature was lower than 5 °C^37,38^. In our study area, the soil temperatures varied from −4 to 10 °C in the summer. However, soil moisture still had an important effect on tundra ER in maritime Antarctica under such low temperatures (Fig. 3a), and a positive correlation (P < 0.05) occurred between Q_10_ and soil moisture (Fig. 3b).

The mean ER at the sites with moss coverage was significantly higher than that at the sites with lichen coverage (Fig. 5a). Generally the mosses grow faster than the lichens, and thus have stronger plant respiration. Previous studies have also demonstrated that CO_2_ emission from terrestrial ecosystems was influenced by the types and distribution of tundra vegetation at King George Island and on the Antarctic Peninsula^11,12^. Therefore vegetation type might be another important factor affecting tundra ER in maritime Antarctica. On the global scale, the ER varied with various vegetation types, and the mean ER rate ranged from 60 mg CO_2_ m^−2^ h^−1^ in the tundra and cryolithozone to 1260 mg CO_2_ m^−2^ h^−1^ in the tropical rainforest^2,19,35^. Different vegetation communities could give rise to differences in CO_2_ emissions through their influence on soil microbial processes^31,36^. The distribution characteristics of tundra vegetation species and the closely related microbial communities are greatly affected by topographical moisture gradients. Lichens (*Usnea sp*.) preferentially occupy higher tundra soil positions and could dominate plant species in relatively tough and exposed environments. Mosses (*Bryum muelenbeckii* and *Bryum pseudotriquetrum*) tend to be found on lower slope positions with high moisture^29^. Wet mosses are characterised by higher biomass and respiration and lower recycling of materials than dry mosses on Signy Island as reported by Davis^39^. Therefore, the role that tundra plants and the related multiple environmental variables play in altering carbon flux cannot be ignored in maritime Antarctica.

In addition, the mean ER at the sites without snow coverage was significantly higher than that at the sites with snow coverage (Fig. 5b), indicating that snow coverage might decrease tundra ER. Snow coverage affects directly or indirectly litter decomposition and carbon release through the effects on soil temperature, water availability, soil fertility, soil microbial activity, and growing-season length^40,41^. Several important researches in the low- and high-arctic tundra ecosystems have shown that ER still occurs at the sites under snow cover, and might provide an important contribution to the annual carbon balance, although snow cover could significantly reduce growing-season ER^7,40,42^. In this study, the interactive effects of soil moisture, soil temperature and snow coverage on ER rates were found (Table 2) and the tundra sites with snow coverage experienced a weak, even extremely low CO_2_ release. The accumulated snow might act as an insulating layer, and inhibit the gas exchange between the underlying tundra soil and the atmosphere^7,41^. Moreover snow melting water increased soil moisture, and might further reduce ER rates^5^. The distribution and coverage of accumulated snow might be important factors affecting the spatial variation of tundra ER in maritime Antarctica.

In general, the synergetic effects among soil moisture, soil temperature, vegetation types and snow coverage on ER rates indicate the complexity of multiple-factors in terms of effects on tundra CO_2_ emissions, inferring that more well-designed field experiments are needed to understand ecosystem processes and the factors affecting ER. In addition, tundra ER rates were obtained from the limited sites during short summertime due to local severe climatic conditions, inconvenient traffic and the manpower absence. Effects of multiple-factors on tundra CO_2_ emissions need long-term multi-site observations to further evaluate the contribution of tundra ER to the annual carbon balance in maritime Antarctica.

## Methods

### Study area

The research area is situated on Fildes Peninsula (61°51′–62°15′S, 57°30′–59°00′W; an area of 30 km^2^) in the southwestern area of King George Island (Fig. 1). Communities of mosses and lichens represent the vegetation on this peninsula^43^. An upland tundra (S62°12′59″, W58°57′52″), which is 500 m away from Great Wall Station, was selected as the study area for the measurements of ecosystem respiration. The upland tundra has the elevation of about 40 m a.s.l. The sampling grounds were covered completely by mosses with the dominant species of *Bryum pseudotriquetrum and Bryum muelenbeckii* or lichens (*Usnea sp*.), and the tundra surface was divided into two distinct areas covered with the mosses and the lichens. The depth of tundra vegetation layer is about 5–10 cm, and under the vegetation cover is an organic clay layer of about 10–15 cm^19^. A more detailed description about the study area was given by Zhu *et al*.^19^.

### Investigation sites and experimental designment

During the summer of 2014/2015, seven sites (GW1-GW7) for the measurements of tundra ER were established along a gentle slope within the upland tundra as shown in Fig. 1: (i) Three observation sites GW1, GW2 and GW3 were equipped with three collars each to test effects of topographic gradient and environmental variables on tundra ER. The sites GW1, GW2 and GW3 were located at the tundra top (about 40 m a.s.l.), the middle slope (about 20 m a.s.l.) and the lower slope (about 10 m a.s.l.), respectively. GW3 was near a lake with the lowest altitude. These three tundra sites GW1–3 were all covered with mosses (*Bryum muelenbeckii* and *Bryum pseudotriquetrum*). The vegetation coverage was about 95% and their height was about 2–3 cm. There were almost no evident differences in the dominant moss species, their height and the phytomass across the sites GW1–3; (ii) The sites GW4 and GW5 were set up in the areas covered with lichens and mosses, respectively, and three sub-sites were selected for each site to study effects of tundra vegetation types on ER. These subsites were almost located at the middle slope (between 15–20 m a.s.l.) on the upland tundra; (iii) The sites GW6 and GW7 (three sub-sites for each site) were established to study effects of accumulated snow coverage on tundra ER. The ground at the site GW6 was covered with 5–6 cm depth accumulated snow, whereas the site GW7 was covered with no snow as the control. These tundra sub-sites were equipped with two collars each, and they were located at the same altitude (about 15 m a.s.l.) with moss vegetation.

### *In situ* ER measurement

ER rates from tundra sites were determined using a CO_2_ flux system (LI-COR Biosciences, Lincoln, NE, USA) with an infrared gas analyzer (model LI-8100A), a 20 cm diameter opaque survey chamber (model LI-8100-103) and a laptop to collect data^44^. The opaque survey chamber was manually mounted on the PVC collar in each plot for the measurement of ER. This system operated at the same time during measurements and CO_2_ concentrations were quantified through optical absorption spectroscopy. The system had been calibrated at the factory using precision gases (CO_2_ gas standards) at controlled temperatures. Polyvinyl chloride (PVC) collars (21.3 cm outer diameter, 20.3 cm inner diameter and 12.5 cm high) were inserted into the soil in the center of each plot at each site, reaching a standard depth of 10 cm. This was done at least 24 h prior to the ER measurements to minimize the effects of the disturbance on CO_2_ emissions. In the snow-covered tundra area, the depth of accumulated snow was only 5–6 cm, and the insertion of 10 cm collar into tundra soil through snow layer was enough to provide the insulation for the measurements of CO_2_ fluxes. During the measuring period, ER rates at each site were based on repetitive measurements over 1.5 min, during which time measurements were made of CO_2_ concentrations inside the chamber at 3 s intervals^44^. The three PVC collars were kept at each site during the whole observation period, and the order of measurements was varied to ensure that the measuring time did not bias the results, resulting in three replicate measurements per site. During the period from Dec 16, 2014 to Feb 2, 2015, the ER rates were measured eight times at the sites GW1, GW2 and GW3. The ER rates at GW4 and GW5 were measured three times on Dec 26, 2014, Jan 12 and Feb 1, 2016. The rates at GW6 and GW7 were also measured three times on Dec 22 and Dec 26, 2014 and Jan 12, 2015. In addition, the diurnal ER measurements were conducted from 2:00 am to 22:00 pm at GW1 and GW2 on Feb 2 and Feb 13, 2015. The measuring time interval was 4 h during the daytime. Normally, ER values are always positive representing CO_2_ emission from the tundra ecosystem to the atmosphere.

### Environmental variables and general soil properties

Soil temperature and soil moisture measurements were conducted at the same time as ER at each site. The monitoring systems consisted of soil temperature probes (Campbell L107E thermocouple, accuracy of ±0.2 °C) and soil moisture probes (CS656 water content reflectometer, accuracy of ±2.5%). These probes were placed at 10 cm depths where PVC soil collars were inserted to determine the ER. All probes were connected to a Campbell Scientific CR 1000 data logger, recording data at every 1 second interval during 1.5 min. Meteorological data, e.g. air temperature (AT), daily sunlight time (ST), total precipitation (TP), and ground temperature (GT) were obtained at the weather station of Great Wall Station.

Soil samples were collected in the plots after ER measurements completed in the summer of 2014/2015. The soils were sampled in a hermetically sealed bag and stored at 4 °C until analyzed. Soil moisture was determined after oven-drying field-moist soil samples to a constant mass at 105 °C (24 hours). Soil samples were homogenized manually, and each 10 g sub-sample (fresh weight) was extracted with 100 ml 1 M KCl solution for one hour and then filtered, and soil NH_4_^+^-N and NO_3_^−^-N concentrations were determined colorimetrically based on Berthelot’s reaction and ion chromatography^13,45^. TOC content was measured by Scheibler’s volumetric method^20^, and TN was quantified on an automatic elemental analyzer (Elementar Vario EL, Hanau, Germany). Soil pH was measured after a 1:3 (soil weight: extractant-volume) dilution of soil with deionized water.

### Data analysis

All statistical analyses were performed using SPSS 20.0 (http://www.spss.com.cn/) and Microsoft Excel 2016 (https://products.office.com/zh-cn/excel) for Windows 10. The univariate exponential function model was used to analyze the relationship between ER and soil temperature according to our previous studies in maritime Antarctica^13^ and the related references^46,47^:

1 $$ER={\beta }_{{0}}{e}^{\beta {1}T}$$

where ER is ecosystem respiration (mg CO_2_ m^−2^ h^−1^), T is mean soil temperature (°C), and β_0_ and β_1_ are constants fitted with the least squares technique^43^. Temperature coefficient (Q_10_ value), an index of temperature dependence^48^, has been widely used to describe the sensitivity of soil respiration to temperature in different types of terrestrial ecosystems as reviewed by Tuomi *et al*.^46^. The Q_10_ value was calculated as:

2 $${Q}_{{10}}={e}^{{10}\beta {1}}$$

The Q_10_ for the whole measurement period (Dec 2014 to Feb 2015) was computed on the basis of the daily averages of respiration rate and soil temperature^13^. The Q_10_ and mean soil temperature, soil moisture during the measurement period were used to examine the relationships between Q_10_ and soil temperature, soil moisture. Exponential regression analyses were used to explore the relationships between ER and soil temperature, soil moisture for all the sites. The single-factor and interaction effects of soil moisture, soil temperature, vegetation types and snow coverage on ER rates were detected using multi-way analysis of variance (ANOVA). Standard error (SE) was used to estimate the uncertainty of the mean of individual fluxes. All the data for ER rates were expressed as mean ± SE. The statistically significant differences between ER means at each type of sites were tested using Fisher’s Least Significant Difference (LSD, P < 0.05) tests. A stepwise multiple regression analysis was used to analyze the multivariate relationships among ER at the sites GW1–3 and soil temperature, soil moisture and other environmental variables.

## Electronic supplementary material

Supplementary Material
