# Supplementary material for: Effects of multiple environmental variables on tundra ecosystem respiration in maritime Antarctica
Source: Sci Rep. 2018 Aug 17;8:12336. doi: 10.1038/s41598-018-30263-6 (PMC6098139; doi:10.1038/s41598-018-30263-6)
Supplement: Supplementary file 1 — Supplementary Material [file 41598_2018_30263_MOESM1_ESM.doc]

**Supplementary Material**

**Effects of multiple environmental variables** **on tundra ecosystem respiration in maritime Antarctica**

Tao Bao^1^, Renbin Zhu^1^*, Xianglan Li^2^, Wenjuan Ye^1^ and Xiao Cheng^2^

*^1^Institute of Polar Environment & Anhui Key Laboratory of Polar Environment and Global Change, School of Earth and Space Sciences, University of Science and Technology of China, Hefei 230026, China.*

*^2^College of Global Change and Earth System Science, Beijing Normal University, Beijing 100875, China.*

**Corresponding author: zhurb@ustc.edu.cn. Tel. 0086-551-63606010, Fax 0086-551-63606010.*

**Figure S1** Relationship between ER and 0-10 cm mean soil temperature at the tundra sites (GW1-7).
